# Modern Technologies Provide New Opportunities for Somatic Hybridization in the Breeding of Woody Plants

**DOI:** 10.3390/plants13182539

**Published:** 2024-09-10

**Authors:** Shuping Liu, Xiaojie Li, Jiani Zhu, Yihong Jin, Chuizheng Xia, Bingsong Zheng, Cristian Silvestri, Fuqiang Cui

**Affiliations:** 1 State Key Laboratory of Subtropical Silviculture, Zhejiang A&F University, Hangzhou 311300, China; 2 Department of Agriculture and Forest Sciences (DAFNE), University of Tuscia, Via San Camillo De Lellis, s.n.c., 01100 Viterbo, Italy

**Keywords:** somatic hybridization, cell fusion, breeding, regeneration, protoplast, woody plants

## Abstract

Advances in cell fusion technology have propelled breeding into the realm of somatic hybridization, enabling the transfer of genetic material independent of sexual reproduction. This has facilitated genome recombination both within and between species. Despite its use in plant breeding for over fifty years, somatic hybridization has been limited by cumbersome procedures, such as protoplast isolation, hybridized-cell selection and cultivation, and regeneration, particularly in woody perennial species that are difficult to regenerate. This review summarizes the development of somatic hybridization, explores the challenges and solutions associated with cell fusion technology in woody perennials, and outlines the process of protoplast regeneration. Recent advancements in genome editing and plant cell regeneration present new opportunities for applying somatic hybridization in breeding. We offer a perspective on integrating these emerging technologies to enhance somatic hybridization in woody perennial plants.

## 1. Introduction

Breeding technologies have evolved from selective breeding and hybridization to contemporary gene-editing approaches [1]. Hybridization, the practice of combining genetic material from different organisms through sexual reproduction, gained scientific prominence following the rediscovery of Mendel’s laws of inheritance in the early 20th century [2]. Traditional hybridization is mostly used in sexually compatible species with relatively short vegetative periods. This constraint restricts the gene pool to those species that can interbreed, thereby limiting genetic diversity and potential improvements. To overcome these limitations, distant hybridization techniques have been developed. Distant hybridization involves crosses between species, genera, or even higher taxonomic ranks, aiming to introduce new genetic material into breeding programs [3,4]. Efforts have also been made to fix hybrid vigor [5,6]. Despite its potential, distant hybridization faces challenges such as hybrid lethality, which is mitigated by embryo rescue techniques that involve in vitro culture to prevent embryo degeneration, though success has been limited to a few species [7].

In the 1960s, the advent of cell and protoplast fusion techniques marked a significant advancement in plant breeding. These methods, collectively known as somatic hybridization, circumvent the limitations of sexual reproduction by allowing for the fusion of cells from different species or genera [8,9]. This approach has proven particularly valuable for woody perennials, which typically have long vegetative periods that make traditional hybridization time-consuming and inefficient. Somatic hybridization provides a means to combine genomes and plastids from different species, offering a potential solution to the limitations of traditional hybridization [10,11].

One prerequisite for successful cell fusion is reducing the negative charge on the phosphate groups of cell membranes. Various strategies have been employed for this purpose, including the use of calcium ions (Ca^2+^), high pH conditions, sodium nitrate (NaNO_3_), polyvinyl alcohol (PVA), and polyethylene glycol (PEG) [12,13]. Techniques such as electrofusion, chemical fusion, and photofusion have been developed to enhance the efficiency and stability of fusion bodies. Among these, PEG-mediated electrofusion has become the most widely used method due to its effectiveness [14,15]. Later, the use of lower PEG concentrations (40% for chemical fusion and 20% for electro-chemical fusion) in combination with direct current pulses has further refined the technique, integrating the advantages of both chemical and electrofusion methods [16].

Somatic hybridization was initially regarded as a highly promising technology due to its potential to combine not only the nuclear genomes but also the plastids of different species [8,16,17]. However, although great efforts have been made to create new species via somatic hybridization, successful combinations are still rare [18]. The primary challenge lies in the low frequency of hybrid regeneration, which may be attributed to issues such as plastid–nucleus incompatibility, inefficient regeneration methods, or developmental lethality [18,19,20]. Even when hybrids are successfully regenerated, they often exhibit sterility and fail to produce viable seeds [19]. This limitation has led to a cyclical pattern of optimism and setbacks for somatic hybridization over the past fifty years [20]. In crops propagated via seeds, embryo failure poses a significant challenge. However, the fact that commercially important woody plants are typically propagated asexually (e.g., through cuttings and micropropagation) offers a potential advantage, as it could circumvent the issue of hybrid sterility. The lack of efficient cell regeneration systems remains a major hurdle for woody plants. Recent advances in cell proliferation and organogenesis research provide new opportunities to optimize regeneration protocols for recalcitrant species, offering promising prospects for the application of somatic hybridization in woody plants.

This review aims to provide a comprehensive evaluation of the progress made in the somatic hybridization of woody plants, highlighting both the limitations and advantages of this breeding approach. We will explore the procedures for regenerating somatic hybrids and discuss how modern breeding technologies can be integrated into somatic hybridization to broaden its application in woody species.

## 2. The Application of Somatic Hybridization in Woody Plants

Woody species, particularly trees, present a unique challenge for breeding due to their long vegetative periods. Traditional breeding methods are often time-consuming and labor-intensive, making somatic hybridization an attractive alternative. This technique facilitates protoplast fusion under in vitro conditions, allowing for the creation of hybrid plants that might not be achievable through sexual reproduction alone [8,9]. Somatic hybridization is particularly valuable for breeding species that are sexually incompatible, asexually propagated, or sterile. By bypassing the sexual phase, somatic hybridization can significantly reduce breeding cycles and introduce new genetic variations into the breeding pool [21]. Unlike traditional hybridization, which typically produces nuclear hybrids while preserving non-inheritable organelle genes, protoplast fusion allows for the transfer and recombination of organelles. This capability promotes plastid evolution, potentially enhancing traits such as product quality, stress resistance, and growth rates [22,23,24]. Thus, somatic hybridization has advantages in promoting plastid evolution and creating desirable plastids. As a result, somatic hybridization has been used to improve various characteristics in plants, including yield, disease resistance, and environmental adaptability [25,26,27].

Citrus species are among the most notable examples of the successful application of somatic hybridization in woody plants. Citrus plants have well-established regeneration methods, making them suitable candidates for somatic hybridization experiments [28,29]. Since the successful regeneration of the first intergeneric Citrus somatic hybrid in 1985, nearly 250 cases of citrus somatic hybrids have been cultivated worldwide, with over 40 cases reported from China alone [30,31]. The ‘leaf protoplasts + embryogenic callus protoplasts’ model has proven effective in Citrus cell fusion, facilitating the selection and regeneration of fused bodies. This model has also played a key role in improving Citrus resilience and fruit quality. Both symmetrical and asymmetrical somatic hybridizations have been employed to produce stress-resistant cultivars, seedless fruits, and disease-resistant varieties [26,32,33]. For example, symmetrical somatic hybridization between Citrus cultivar species and *Poncirus trifoliata* has been conducted to produce stress-resistant cultivars or ideal rootstocks [33,34,35,36]. Generally, symmetrical somatic hybridization has been widely used for rootstock breeding, as undesirable traits can be incorporated into the fused product. However, with asymmetric hybridization, the situation can be very different. Asymmetrical somatic hybridization has been conducted in Citrus to obtain male-sterile trees, seedless fruits, and disease-resistant cultivars [37,38,39]. Many somatic hybrid Citrus cultivars have been commercialized. Typical cases that have been published are summarized in Table 1.

Beyond Citrus, efforts to apply somatic hybridization to other woody species have yielded some success. Early cases included cherry, pear, persimmon, Passiflora species, and apple [40,41,42,43,44]. More recent progress has been made. Hybrid cells have been achieved in species such as mango, mulberry, jujube, and rose [22,45,46,47,48]. Despite these advances, the regeneration of fused cells—the final step of somatic hybridization, which determines the success of somatic hybridization breeding—remains a critical challenge. For instance, mulberry hybrid cells were successfully obtained in the 1980s but failed to develop into plants [47]. Woody species’ failure to regenerate could explain why cell fusion technology has mainly succeeded in herbaceous species, as these have more sophisticated regeneration systems [49,50]. Recent achievements in protoplast culturing have been reported in species like grape [51], litchi [52], olive [53], camellia [54], Jasminum [55], pecan [56], apricot [57], poplar [58], and various gymnosperms [59,60,61], providing very good preconditions for the application of somatic hybridization. However, issues such as the instability of fusion bodies and abnormalities in embryo development continue to pose challenges [62]. Establishing robust regeneration systems is crucial for advancing somatic hybridization in woody species.

**Table 1:** Protocol for woody species with successful protoplast fusion.

Category	Latin Name	Improvement in Agronomical Characteristics	References
Pear/cherry	*Pyrus communis* var. *pyraster* L. × *Prunus avium* × *pseudocerasus*	Enhanced traits such as chromosome number, morphological characteristics, and leaf isozyme profiles	[40]
Persimmon	*Diospyros glandulosa* × *D. kaki*	Resolved the hybridization barrier in *D. kaki*	[41]
Mango	*Mangifera indica* L.	Hybrids between cultivars	[46]
*Passiflora*	*Passiflora edulis* f. *flavicarpa* Degener. × *P. cincarnata* L.		[42]
	*Passiflora edulis* f. *flavicarpa* Degener. × *P. amethystina* Mikan		[43]
*Citrus*	*Citrus reshni* Hort. Ex Tan × winter Haven citrumelo *Citrus reshni Hort.* Ex Tan × *citrange* *Citurs sinensis* L. Osbeck × (*C.* × *paradisi* × *Poncirus. trifoliata*)	Promote high fruit quality and good yields	[35]
	*Citrus australasica* F. Muell × *Citrus sinensis* *Citrus australasica* F. Muell × *Citrus ‘Ju You’*	Perceive tolerance to Huanglongbing (HLB)	[33]
	*Citrus japonica Thunb* × *Citrus paradisi Macfad*	Enhancing resistance to Citrus canker	[39]
	*Citrus deliciosa Ten* × *Poncirus trifoliata* (L.) Raf	Improving natural chilling and light stress tolerances	[63]
	*Citrus latipes* × W. Murcott	Improving resistance to greening disease and reducing seedlessness	[64]
	*Citrus reticulata* × *Citrus paradisi*	Improving fruit characteristics	[65]
	*Citrus unshiu* Marc. × *Citru sinensis* L. Osb.	CMS	[66]
	*Citrus sinensis* L. Osbeck × *Citrus limon* L. Burm	Improving fruit oil quality	[67]
	*Citrus sinensi s* × [*Citrus reticulata* × (*Citrus paradisi* × *Citrus reticulata*)]	Improving citrus scion	[68]
	*Citrus sinensis* L. × *Poncirus trifoliata* L.	Addressing the threats posed by abiotic constraints	[69]
	*Citrus unshiu* Marc. × [ *Citrus reticulata Blanco* × (*Citrus reticulata Blanco* × *Citrus paradisi Macf*)]	CMS	[70]
	*Citrus sinensis* × *Citrus* *deliciosa*	Improving the tolerance to *Xanthomonas axonopodis* pv. *citri* and *Xylella fastidiosa*	[71]
	*Citrus medica* L. × *C. limon* (L.) Burm. F.	Improving acidity	[72]
	*Citrus unshiu* × *Citrus grandis*	CMS	[37]
	*Citrus limonia* L. Osbeck × *Citrus aurantium* L.	Improving blight and CTV resistance	[73]

## 3. Protoplast Regeneration: A Key Step in Somatic Hybridization

Protoplast regeneration is a critical step in somatic hybridization and often follows methods developed for herbaceous plants. This process typically involves the use of growth regulators such as auxins and cytokinins to stimulate genome reprogramming and promote cell differentiation [74]. However, most regeneration methods were initially developed for tissue or organ propagation rather than single-cell regeneration. Effective regeneration from protoplasts requires the development of cellular conditions similar to zygotic embryos, a process known as de novo organogenesis or somatic embryogenesis [75]. Over recent decades, many culture recipes have been invented to cultivate corresponding plant species [76,77]. More than 46 woody genotypes have been regenerated from protoplasts in the last century, representing 32 species, 18 genera, and 12 families [78]. The factors influencing protoplast regeneration will be discussed below.

The isolation of protoplasts is a crucial factor for successful regeneration [79,80]. This process involves the degradation of the cell wall using enzymes and maintaining protoplast turgor with osmotic regulators [81]. Protoplasts can be isolated from leaves, cotyledons, roots, petioles, hypocotyls, petals, calli, and suspension cultures. Regardless of the tissue type, the condition of the plant materials is fundamental for subsequent regeneration. Generally, juvenile tissues are considered more amenable to regeneration [79,82]. Factors such as plant growth conditions, tissue pretreatment, and enzyme and buffer composition significantly impact protoplast yield and viability [83]. Tissue pretreatments such as physical disruption (e.g., slicing), vacuum infiltration of the enzyme solution, or pre-plasmolysis treatment will significantly improve protoplast yield (revised in [84]). Multiple cell wall-degrading enzymes (cellulases, beta-glucanases, xylanases, protopectinases, polygalacturonases, pectin lyases, and pectinesterases) have been used, and a mixture of Cellulase R-10, Macerozyme R-10, and Pectolyase Y-23 is currently the most-used enzyme recipe [84,85]. The buffer solution for enzymolysis usually contains osmolytes (KCl, CaCl_2_, mannitol, sorbitol, or salts), pH buffer (MES, ethanesulfonic acid), reducing agent (β-mercaptoethanol), and enzyme-protecting agents (bovine serum albumin, BSA). Fine-tuning the buffer recipes is necessary for optimal enzyme activity. Additionally, temperature is a factor impacting enzyme activity [84,85].

The digestion period varies between species and enzyme concentrations. Typically, periods ranging from 1 to 18 h have been reported in the literature, with 2 and 4 h being the most common [86]. Both the concentration of enzymes and the duration of digestion have significant impacts on protoplast regeneration. When all conditions are optimized, cultivation in the dark has been reported to further improve protoplast regeneration ability [83]. After optimized digestion, protoplasts need to be purified to remove cell wall debris and undigested materials. This process eliminates negative effects on cell division and development [61].

The culture medium used for protoplast regeneration is also a key factor. A suitable culture medium will enhance regeneration frequency. Commercial media, such as WPM, MS, Gamborg B5, KM, Y3, and Nitsch, are usually initially tested for the species used in somatic hybridization. Then, the recipes are optimized according to the specific nutrient requirements of each species [87]. The carbon supplements also vary between species. Usually, 1–3% sucrose is used [79]. Osmotic pressure, provided by chemicals like mannitol, sorbitol, and sucrose, is essential for hybrid cell regeneration [49]. Mannitol is the most common. However, some species prefer myo-inositol or other osmotic agents [88]. During the cultivation of hybrid cells, the cell wall will re-form. Thus, osmolarity needs to be gradually decreased. Growth regulators are essential for plant regeneration. To establish a regeneration system, multiple types of hormones will be screened, including cytokinins and auxins [84]. Typically, once the types of plant-responsive cytokinins and auxins are determined, the concentrations of these two hormones need to be finely adjusted to optimize the efficiency of regeneration. Additionally, gibberellic acid is necessary for the regeneration of some cultivated calli [89], and the ratio between auxin and abscisic acid is especially important for the regeneration of gymnosperms [90].

Even under optimized conditions, many woody plants are still recalcitrant to regeneration. Thus, multiple supplements have been added to promote regeneration. Reactive oxygen species (ROS), phenolic compounds, and ethylene are considered the major negative effectors of plant regeneration [91]. Therefore, antioxidants (ascorbic acid, citric acid, reduced glutathione, and L-cysteine), phenolic absorption materials (such as polyvinylpyrrolidone and activated charcoal), and ethylene inhibitors (silver nitrate) are often used to mitigate these effects [92,93,94].

The initial phase of protoplast regeneration involves culturing a single fused cell into a multicellular cluster. In somatic hybridization breeding, it is crucial to develop the callus from a single hybrid cell to achieve a homozygous line, making it essential to prevent contact between fused cells. To minimize cell aggregation, fused cells are typically cultured on semi-solid media at a low density rather than in liquid media [84,95,96]. These cells are then microscopically screened within a few days of cultivation to identify desirable hybrid candidates. Research into poplars has shown that stress-free conditions along with contact with a solid surface, promote the successful development of protoplasts into multicellular structures [80]. Overall, regeneration is a pivotal process in somatic hybridization. Citrus species have produced the most cultivars through somatic hybrid breeding (Table 1), owing much of their success to highly efficient regeneration systems [29,97]. Therefore, media with precisely optimized hormone levels must be meticulously prepared for each stage of protoplast regeneration.

## 4. Limitations of Somatic Hybridization in Woody Plants

The process of somatic hybridization in plants encompasses three primary stages: protoplast isolation, fusion, and the propagation/regeneration of hybrids. Correspondingly, the limitations associated with somatic hybridization breeding can be delineated into three categories: the genotype-dependent nature of protoplast isolation and hybrid regeneration, the occurrence of sterile hybrids, and the low survival rates observed in hybrid progeny [81,98,99,100]. While protocols for isolating protoplasts from woody plants mirror those used for herbaceous species, the unique characteristics of woody plant cell walls and protoplasts render cell wall degradation and protoplast isolation less efficient and less well established [101,102]. Factors such as tissue type, enzymatic composition, and solution concentrations play pivotal roles; however, protoplast yield is also highly contingent on plant genotypes [100,103]. For instance, successful protoplast isolation has eluded varieties like *Rosa indica*, *R. multiflora*, and *R. corymbifera* cultivars ‘Laxa’ and ‘Elina’, even after more than four decades since the advent of cell fusion techniques [104].

Another significant hurdle in the somatic hybridization of woody plants is the generally low survival and regeneration rates of hybrids. Many woody species lack established in vitro cultivation methodologies [105], and there exists substantial genetic variability among tree and shrub species concerning cell differentiation and regeneration [105,106,107]. Only a select few species, such as those within the *Citrus* and *Vaccinium* genera, possess well developed regeneration protocols [29,108], thereby limiting the applicability of somatic hybridization. Even in species with mature and stable cell fusion technologies, like Citrus, success rates remain modest. For example, Xiao et al. generated over 100 embryoids via electrofusion in Citrus but successfully developed and transplanted only 12 plants [66]. The polyploid nature of hybrids contributes to low survival rates; in persimmon, both octoploid and hexaploid lines were produced through cell fusion, yet only some octoploid lines matured into healthy plants [41]. Sterility is a common issue in symmetric protoplast fusion in woody plants, where quantitative data are scarce. In herbaceous plants, for example, one-third of the hybrid progeny resulting from the fusion between *Brassica napus* cv. Zhongshuang4 and its wild relative *Sinapis arvensis* were sterile [109]. The underlying causes of sterility in heterozygous hybrids remain inadequately understood. Hybrids derived from parent plants with unequal ploidy levels are typically sterile [110], and asymmetric protoplast fusion can result in incompatible plastids within hybrid cells, also leading to sterility [107]. Currently, effective solutions to address sterility are lacking.

Nevertheless, woody plants possess an inherent ability for asexual propagation both in natural settings and cultivation environments. Consequently, seed production in somatic hybrids of woody plants is less critical than in annual species. Thus, the regeneration of hybrid cells could serve as the culminating step in the somatic hybridization breeding of woody plants. Despite this, protoplast regeneration remains challenging for most woody species. Establishing a regeneration system for recalcitrant species using traditional methodologies is not only exceedingly laborious but also fraught with difficulty, thereby impeding the broader application of somatic hybridization in woody plants.

## 5. Current Plant Regeneration Methods and Their Potential for Tree Breeding with Somatic Hybridization

Currently, multiple novel approaches are under investigation to enhance plant regeneration, with some demonstrating genotype-independent effects [111]. Plant regeneration, which involves extensive genome reprogramming, can be stimulated by various pioneer genes. Optimized protocols (such as specific culture media and appropriate hormone concentrations) combined with the introduction of regeneration-promoting genes have significantly improved protoplast regeneration, even in species that are typically difficult to regenerate [111]. It has been observed that hormones commonly used in tissue culture, such as auxin and cytokinin, enhance plant regeneration by modulating the expression of key genes like *WUSCHEL* (*WUS*) and *BABY BOOM* (*BBM*) [112]. Expression of these genes in somatic cells, either individually or in combination, is often sufficient to initiate whole plant regeneration or shoot formation [113]. Additionally, genes associated with auxin/cytokinin biosynthesis, injury responses, and cell-fate determination, such as IPTs, glutamate receptor-like proteins, STMs, and WOXs, have also been successfully employed to promote plant regeneration [114]. However, the constitutive overexpression of these genes often disrupts normal plant development and fertility. To address this, three strategies have been developed: the use of inducible promoters to control gene expression, the Cre/loxP system for cassette removal post-regeneration, and non-integrating methods [115]. Inducible promoters have been particularly successful in inducing regeneration across multiple woody species [116]. While Agrobacterium-mediated transformation has been the primary method, protoplast-mediated transformation offers a promising alternative for similar regenerative outcomes.

Recent research has identified genes with minimal impact on overall plant development, such as *WOX5*, *GRF4*, and *GIF1*, which show greater potential in promoting regeneration. The GRF complexes have been applied in various crops with promising results [113,117,118]. In Citrus, two negative regulators of somatic embryogenesis were identified, and the knockdown of these regulators significantly enhanced somatic embryogenesis [119]. As RNA interference (RNAi) can be delivered via viruses without altering the plant genome [120], these negative regulators hold potential for advancing hybrid cell regeneration. Ongoing research continues to explore growth-regulating genes in shrub and tree species, with new genes being identified that help overcome regeneration barriers, thereby advancing somatic hybridization as a viable breeding method.

The CRISPR-Cas system, derived from a bacterial immune mechanism, has been widely adopted in gene editing for plant breeding due to its ability to make precise DNA cuts guided by designed RNA sequences [121,122,123]. While it is theoretically feasible to use CRISPR-Cas9 to knock out genes that inhibit protoplast regeneration, the permanent loss of these genes could adversely affect normal plant growth. To mitigate this, the CRISPR-Cas9 system has been adapted into an RNA-guided platform for sequence-specific gene regulation. In this modified version, Cas9 is rendered catalytically inactive but retains its ability to bind specific DNA sequences, allowing it to repress or activate nearby gene expression when coupled with a strong activator [124]. The CRISPR-Combo system, which integrates CRISPR-Cas9 gene editing with MCP SunTag activator-mediated gene activation, allows for simultaneous editing and activation of regeneration-promoting genes [125,126]. This technology has been applied in poplar and offers the advantage of flexibility and multiplexing, enabling the testing of various combinations of endogenous morphogenic transcription factors (MTFs) [125,127]. When combined with the inducible LexA-VP16-ER system, CRISPR-Combo can be activated at specific times to enhance regeneration [128]. Consequently, CRISPR technology not only facilitates precise genome editing but also supports the editing of genes to enhance the stability and developmental potential of cell fusion bodies [129]. The theoretical framework for single-cell regeneration is now well established [130,131], and these advancements hold promise for accelerating the development of regeneration systems in tree and shrub species, thereby expanding the potential applications of somatic hybridization in these plants.

## 6. Integrating Precise Gene Editing with Protoplast Fusion: A Promising Breeding Strategy

Gene editing provides a precise approach to plant breeding [132,133], while cell fusion allows for the recombination of entire chromosomes between cells. The integration of these two methods holds great potential for advancing breeding strategies, though it requires the development of robust gene transformation and efficient regeneration systems. However, gene transformation is highly genotype-dependent, with significant variability in transformation efficiency, even among plants of the same species [51,134]. For instance, in commercial crops where regeneration systems may be well established for one cultivar, other cultivars can still prove resistant to Agrobacterium-mediated gene transformation [111].

**Figure 1:**
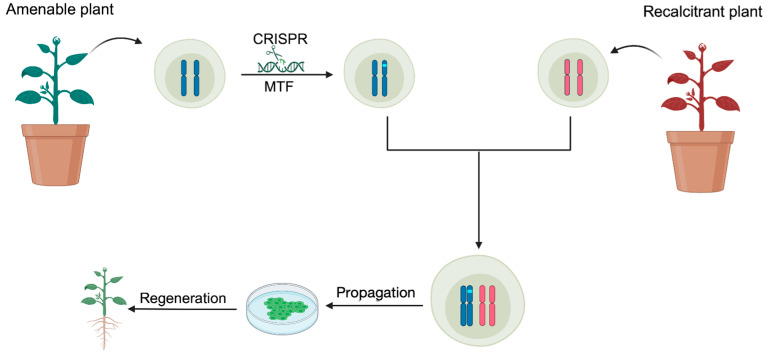
Strategies for application of current breeding biotechnologies in gene transformation-recalcitrant plants via somatic hybridization. The plant that is amenable to gene transformation serves as an intermediate to incorporate gene cassettes. Somatic hybridization combines the two genomes with the gene cassettes. Gene cassettes play roles such as gene editing and regeneration promotion in the hybrids.

Somatic hybridization, which involves protoplasts, offers an alternative by facilitating Agrobacterium-independent transformation through direct plastid transformation. This method has already yielded DNA-free, edited plants in several woody species via protoplast transformation [51,84,135]. This approach allows for the seamless integration of transgene-free gene editing with somatic hybridization. Alternatively, cell fusion can act as an intermediary step. For example, gene-editing cassettes can first be introduced into varieties amenable to gene transformation, and then the edited cells can be fused with those from recalcitrant varieties (Figure 1). This strategy is particularly advantageous for species with long vegetative phases, such as shrubs and trees, where introducing cassettes through sexual crosses is time-intensive. In addition to gene editing, cassettes containing regeneration-promoting genes can also be applied using this strategy.

Recent advancements in CRISPR technology, along with morphogenic transcription factor (MTF)-assisted transformation methods, show significant potential in overcoming genotype dependency [113]. By introducing CRISPR-associated regulators or MTFs into transformation-amenable varieties and subsequently fusing them with recalcitrant ones, regeneration can be promoted, enhancing the overall efficiency of breeding programs.

Somatic hybridization is recognized as a biosafe breeding method. Integrating modern techniques, such as gene editing and somatic hybridization, offers a promising approach for breeding woody plants. However, this integration raises concerns regarding the use of genetically modified organisms (GMOs) and CRISPR technology in somatic hybrid plants. Biosafety issues surrounding transgenic plants may present barriers to the adoption of somatic hybrids that incorporate gene transformation [136]. The CRISPR-Cas system, however, enables gene editing without the integration of foreign DNA into the plant genome [137]. Specifically, transgene-free strategies, including the transient expression of CRISPR vectors or the use of Cas protein-guide RNA complexes, can alleviate concerns related to GMOs [51,84,135]. Despite these advancements, the issue of off-target effects in CRISPR-Cas systems necessitates the further refinement of CRISPR technology [138].

In conclusion, somatic hybridization is a well-established breeding method that has been enhanced by recent technological advancements. While current technologies that reduce genotype dependency in regeneration and transformation have not yet been fully integrated into somatic hybridization, they hold significant promise for their application in modern breeding. Somatic hybridization, with its ability to combine genetic material across species and genera, remains an irreplaceable breeding method. This review highlights the development of cell fusion techniques in breeding and underscores their potential when combined with contemporary breeding technologies.

## References

[1] Darkwa K., Olasanmi B., Asiedu R., Asfaw A. (2020). Review of Empirical and Emerging Breeding Methods and Tools for Yam (*Dioscorea* spp.) Improvement: Status and Prospects. Plant Breed..

[2] Lamichhane S., Thapa S. (2022). Advances from Conventional to Modern Plant Breeding Methodologies. Plant Breed. Biotechnol..

[3] Ahmar S., Gill R.A., Jung K.-H., Faheem A., Qasim M.U., Mubeen M., Zhou W. (2020). Conventional and Molecular Techniques from Simple Breeding to Speed Breeding in Crop Plants: Recent Advances and Future Outlook. Int. J. Mol. Sci..

[4] Goulet B.E., Roda F., Hopkins R. (2017). Hybridization in Plants: Old Ideas, New Techniques [OPEN]. Plant Physiol..

[5] Liu C., Wang J., Lu H., Huang Y., Yan H., Liang H., Wang C., Wang K. (2024). Engineering Synthetic Apomixis in Different Hybrid Rice Varieties Using the Fix Strategy. New Crops.

[6] Xu N., Yu Z., Wang X., Lu J., Chen H., Sun Q., Fei C., Cui X., Xu Z., Xu Q. (2024). Influence of Natural and Artificial Selection on the Yield Differences among Progeny Derived from Crossing between Subspecies in Cultivated Rice. New Crops.

[7] Uma S., Lakshmi S., Saraswathi M.S., Akbar A., Mustaffa M.M. (2011). Embryo Rescue and Plant Regeneration in Banana (*Musa* spp.). Plant Cell Tissue Organ Cult..

[8] Cocking E.C. (1960). A Method for the Isolation of Plant Protoplasts and Vacuoles. Nature.

[9] Takebe I., Labib G., Melchers G. (1971). Regeneration of Whole Plants from Isolated Mesophyll Protoplasts of Tobacco. Naturwissenschaften.

[10] Grosser J.W., Ollitrault P., Olivares-Fuster O. (2000). Somatic Hybridization in Citrus: An Effective Tool to Facilitate Variety Improvement. Vitr. Cell. Dev. Biol.-Plant.

[11] Mohammadi M.A., Wai M.H., Rizwan H.M., Qarluq A.Q., Xu M., Wang L., Cheng Y., Aslam M., Zheng P., Wang X. (2023). Advances in Micropropagation, Somatic Embryogenesis, Somatic Hybridizations, Genetic Transformation and Cryopreservation for Passiflora Improvement. Plant Methods.

[12] Bates G.W. (1985). Electrical Fusion for Optimal Formation of Protoplast Heterokaryons in Nicotiana. Planta.

[13] Begna T. (2020). Review on Somatic Hybridization and Its Role in Crop Improvement. J. Biol. Agric. Healthc..

[14] Nahirñak V., Almasia N.I., González M.N., Massa G.A., Décima Oneto C.A., Feingold S.E., Hopp H.E., Vazquez Rovere C. (2022). State of the Art of Genetic Engineering in Potato: From the First Report to Its Future Potential. Front. Plant Sci..

[15] Borgato L., Conicella C., Pisani F., Furini A. (2007). Production and Characterization of Arboreous and Fertile Solanum Melongena + Solanum Marginatum Somatic Hybrid Plants. Planta.

[16] Bashir T., Mishra R., Hasan D.-M., Mohanta T., Bae H. (2018). Effect of Hybridization on Somatic Mutations and Genomic Rearrangements in Plants. Int. J. Mol. Sci..

[17] Flick C.E., Bravo J.E., Evans D.A. (1983). Organelle Segregation Following Plant Protoplast Fusion. Trends Biotechnol..

[18] Sigareva M.A., Earle E.D. (1997). Direct Transfer of a Cold-Tolerant Ogura Male-Sterile Cytoplasm into Cabbage (*Brassica Oleracea* Ssp. Capitata) via Protoplast Fusion. Theor. Appl. Genet..

[19] Anushma P.L., Dhanyasree K., Rafeekher M. (2021). Wide Hybridization for Fruit Crop Improvement: A Review. Int. J. Chem. Stud..

[20] Holmes M. (2018). Somatic Hybridization: The Rise and Fall of a Mid-Twentieth-Century Biotechnology. Hist. Stud. Nat. Sci..

[21] Shuro A. (2018). Review Paper on the Role of Somatic Hybridization in Crop Improvement. Int. J. Res..

[22] Khan G., Shukla P., Ravindra M., Gani A., Shabnam Y., Srinivasulu S., Sharma, Rohela G. (2016). Somatic Hybridization as a Potential Tool for Mulberry Improvement: A Review. Ind. Horti. J..

[23] Cho K.-S., Lee H.-O., Lee S.-C., Park H.-J., Seo J.-H., Cho J.-H., Park Y.-E., Choi J.-G., Yang T.-J. (2022). Mitochondrial Genome Recombination in Somatic Hybrids of *Solanum Commersonii* and *S. Tuberosum*. Sci. Rep..

[24] Yu Y., Yu P.-C., Chang W.-J., Yu K., Lin C.-S. (2020). Plastid Transformation: How Does It Work? Can It Be Applied to Crops? What Can It Offer?. Int. J. Mol. Sci..

[25] Cai X., Duan Y., Fu J., Guo W. (2010). Production and Molecular Characterization of Two New Citrus Somatic Hybrids for Scion Improvement. Acta Physiol. Plant.

[26] Guo W.-W., Xiao S.-X., Deng X.-X. (2013). Somatic Cybrid Production via Protoplast Fusion for Citrus Improvement. Sci. Hortic..

[27] Wang W., Xu J., Fang H., Li Z., Li M. (2020). Advances and Challenges in Medicinal Plant Breeding. Plant Sci..

[28] Bhat S.R., Chitralekha P., Chandel K.P.S. (1992). Regeneration of Plants from Long-Term Root Culture of Lime, *Citrus aurantifolia* (Christm.) Swing. Plant Cell Tissue Organ Cult..

[29] Singh S., Rajam M.V. (2010). Highly Efficient and Rapid Plant Regeneration in *Citrus sinensis*. J. Plant Biochem. Biotechnol..

[30] Ohgawara T., Kobayashi S., Ohgawara E., Uchimiya H., Ishii S. (1985). Somatic Hybrid Plants Obtained by Protoplast Fusion between *Citrus sinensis* and *Poncirus trifoliata*. Theor. Appl. Genet..

[31] Guo W.W., Deng X.X. (2001). Wide Somatic Hybrids of Citrus with Its Related Genera and Their Potential in Genetic Improvement. Euphytica.

[32] Ollitrault P., Germanà M.A., Froelicher Y., Cuenca J., Aleza P., Morillon R., Grosser J.W., Guo W., Gentile A., La Malfa S., Deng Z. (2020). Ploidy Manipulation for Citrus Breeding, Genetics, and Genomics. The Citrus Genome.

[33] Dutt M., Mahmoud L.M., Chamusco K., Stanton D., Chase C.D., Nielsen E., Quirico M., Yu Q., Gmitter F.G., Grosser J.W. (2021). Utilization of Somatic Fusion Techniques for the Development of HLB Tolerant Breeding Resources Employing the Australian Finger Lime (*Citrus australasica*). PLoS ONE.

[34] Abbate L., Panno S., Mercati F., Davino S., Fatta Del Bosco S. (2019). Citrus Rootstock Breeding: Response of Four Allotetraploid Somatic Hybrids to Citrus Tristeza Virus Induced Infections. Eur. J. Plant Pathol..

[35] Dambier D., Barantin P., Boulard G., Costantino G., Mournet P., Perdereau A., Morillon R., Ollitrault P. (2022). Genomic Instability in Somatic Hybridization between Poncirus and Citrus Species Aiming to Create New Rootstocks. Agriculture.

[36] Mahmoud L.M., Killiny N., Holden P., Gmitter F.G., Grosser J.W., Dutt M. (2023). Physiological and Biochemical Evaluation of Salt Stress Tolerance in a Citrus Tetraploid Somatic Hybrid. Horticulturae.

[37] Guo W.W., Prasad D., Cheng Y.J., Serrano P., Deng X.X., Grosser J.W. (2004). Targeted Cybridization in Citrus: Transfer of Satsuma Cytoplasm to Seedy Cultivars for Potential Seedlessness. Plant Cell Rep..

[38] Jiang N., Feng M.-Q., Cheng L.-C., Kuang L.-H., Li C.-C., Yin Z.-P., Wang R., Xie K.-D., Guo W.-W., Wu X.-M. (2023). Spatiotemporal Profiles of Gene Activity in Stamen Delineate Nucleo-Cytoplasmic Interaction in a Male-Sterile Somatic Cybrid Citrus. Hortic. Res..

[39] Murata M.M., Omar A.A., Mou Z., Chase C.D., Grosser J.W., Graham J.H. (2019). Novel Plastid-Nuclear Genome Combinations Enhance Resistance to Citrus Canker in Cybrid Grapefruit. Front. Plant Sci..

[40] Ochatt S.J., Patat-Ochatt E.M. (1995). Protoplast Technology for the Breeding of Top-Fruit Trees (Prunus, Pyrus, Malus, Rubus) and Woody Ornamentals. Euphytica.

[41] Tamura M., Tao R., Sugiura A. (1998). Production of Somatic Hybrids between Diospyros Glandulosa and D. Kaki by Protoplast Fusion. Plant Cell Tissue Organ Cult..

[42] Otoni W.C., Blackhall N.W., d’Utra Vaz F.B., Casali V.W., Power J.B., Davey M.R. (1995). Somatic Hybridization of the Passiflora Species, *P. edulisf. Flavicarpa* Degener. and *P. incarnata* L. J. Exp. Bot..

[43] Barbosa L.V., Vieira M.L.C. (1997). Meiotic Behavior of Passion Fruit Somatic Hybrids, Passiflora Edulis f. Flavicarpa Degener + P. Amethystina Mikan. Euphytica.

[44] Huancaruna-Perales E., Schieder O., Hanke V. Investigation on Somatic Hybridization in Apple. Proceedings of the Eucarpia Symposium on Fruit Breeding and Genetics.

[45] Schum A., Hofmann K., Felten R. (2002). Fundamentals for Integration of Somatic Hybridization in Rose Breeding. Acta Hortic..

[46] Rezazadeh R., Williams R.R., Harrison D.K. (2011). Factors Affecting Mango (*Mangifera indica* L.) Protoplast Isolation and Culture. Sci. Hortic..

[47] Chanotra S. (2019). Role of Biotechnology in Mulberry Improvement. J. Pharmacogn. Phytochem..

[48] Ahmad I., Maryam, Ercisli S., Anjum M.A., Ahmad R. (2023). Progress in the Methods of Jujube Breeding. Erwerbs-Obstbau.

[49] Eeckhaut T., Lakshmanan P.S., Deryckere D., Van Bockstaele E., Van Huylenbroeck J. (2013). Progress in Plant Protoplast Research. Planta.

[50] Wang J., Jiang J., Wang Y. (2013). Protoplast Fusion for Crop Improvement and Breeding in China. Plant Cell Tissue Organ Cult..

[51] Scintilla S., Salvagnin U., Giacomelli L., Zeilmaker T., Malnoy M.A., Rouppe van der Voort J., Moser C. (2022). Regeneration of Non-Chimeric Plants from DNA-Free Edited Grapevine Protoplasts. Front. Plant Sci..

[52] Yu C., Chen Z., Lu L., Lin J. (2000). Somatic Embryogenesis and Plant Regeneration from Litchi Protoplasts Isolated from Embryogenic Suspensions. Plant Cell Tissue Organ Cult..

[53] Sahouli S., Abdeddaim K.K., Werbrouck S.P.O. (2022). Isolation, Chemical Fusion, and Culture Systems for Olive (*Olea Europaea* L.) Protoplasts. Vitr. Cell. Dev. Biol.-Plant.

[54] Li S., Zhao R., Ye T., Guan R., Xu L., Ma X., Zhang J., Xiao S., Yuan D. (2022). Isolation, Purification and PEG-Mediated Transient Expression of Mesophyll Protoplasts in Camellia Oleifera. Plant Methods.

[55] Ahmed M.A., Miao M., Pratsinakis E.D., Zhang H., Wang W., Yuan Y., Lyu M., Iftikhar J., Yousef A.F., Madesis P. (2021). Protoplast Isolation, Fusion, Culture and Transformation in the Woody Plant *Jasminum* spp. Agriculture.

[56] Casales F.G., Van der Watt E., Coetzer G.M. (2018). Propagation of Pecan (*Carya illinoensis*): A Review. Afr. J. Biotechnol..

[57] Ortín-Párraga F., Burgos L. (2003). Isolation and Culture of Mesophyll Protoplast from Apricot. J. Hortic. Sci. Biotechnol..

[58] Wang Y., Huang M.R., Wei Z.M., Sun Y.R., Chen D.M., Xu Z.H., Zhang L.M., Xu N. (1995). Regeneration of Simon Poplar (*Populus simonii*) from Protoplast Culture. Plant Cell Rep..

[59] Faye M., David A. (1983). Isolation and Culture of Gymnosperm Root Protoplasts (*Pinus pinaster*). Physiol. Plant..

[60] David A., Bonga J.M., Durzan D.J. (1987). Conifer Protoplasts. Cell and Tissue Culture in Forestry.

[61] Castelblanque L., García-Sogo B., Pineda B., Moreno V. (2010). Efficient Plant Regeneration from Protoplasts of Kalanchoe Blossfeldiana via Organogenesis. Plant Cell Tissue Organ Cult..

[62] Pais M.S. (2019). Somatic Embryogenesis Induction in Woody Species: The Future After OMICs Data Assessment. Front. Plant Sci..

[63] Oustric J., Morillon R., Ollitrault P., Herbette S., Luro F., Froelicher Y., Tur I., Dambier D., Giannettini J., Berti L. (2018). Somatic Hybridization between Diploid Poncirus and Citrus Improves Natural Chilling and Light Stress Tolerances Compared with Equivalent Doubled-Diploid Genotypes. Trees.

[64] Altaf S. (2016). Somatic Hybridization for Citus Scion and Rootstock Improvement against Greening Disease and for Seedlessness. Thesis.

[65] Satpute A.D., Chen C., Gmitter F.G., Ling P., Yu Q., Grosser M.R., Grosser J.W., Chase C.D. (2015). Cybridization of Grapefruit with ‘Dancy’ Mandarin Leads to Improved Fruit Characteristics. J. Am. Soc. Hortic. Sci..

[66] Xiao S.-X., Biswas M.K., Li M.-Y., Deng X.-X., Xu Q., Guo W.-W. (2014). Production and Molecular Characterization of Diploid and Tetraploid Somatic Cybrid Plants between Male Sterile Satsuma Mandarin and Seedy Sweet Orange Cultivars. Plant Cell Tissue Organ Cult..

[67] Fatta Del Bosco S., Abbate L., Tusa N., Strano T., Renda A., Ruberto G. (2013). Genetic Improvement of *Citrus* Fruits: The Essential Oil Profiles in a *Citrus limon* Backcross Progeny Derived from Somatic Hybridization. Food Res. Int..

[68] Soriano L., de Assis Alves Mourão Filho F., Camargo L.E.A., Cristofani-Yaly M., Latado R.R., de Andrade Pacheco C., de Azevedo F.A., Mendes B.M.J. (2012). Regeneration and Characterization of Somatic Hybrids Combining Sweet Orange and Mandarin/Mandarin Hybrid Cultivars for Citrus Scion Improvement. Plant Cell Tissue Organ Cult..

[69] Dambier D., Benyahia H., Pensabene-Bellavia G., Aka Kaçar Y., Froelicher Y., Belfalah Z., Lhou B., Najat H., Printz B., Morillon R. (2011). Somatic Hybridization for Citrus Rootstock Breeding: An Effective Tool to Solve Some Important Issues of the Mediterranean Citrus Industry. Plant Cell Rep..

[70] Cai X., Fu J., Chen C., Guo W. (2009). Cybrid/Hybrid Plants Regenerated from Somatic Fusions between Male Sterile Satsuma Mandarin and Seedy Tangelos. Sci. Hortic..

[71] Pavan A., Calixto M.C., Cardoso S.C., Mendes B.M.J., Filho A.B., Lopes J.R.S., de Carvalho C.R., Filho F.d.A.A.M. (2007). Evaluation of ‘Hamlin’ Sweet Orange + ‘Montenegrina’ Mandarin Somatic Hybrid for Tolerance to *Xanthomonas axonopodis* Pv. *Citri* and *Xylella fastidiosa*. Sci. Hortic..

[72] Viloria Z., Grosser J.W. (2005). Acid Citrus Fruit Improvement via Interploid Hybridization Using Allotetraploid Somatic Hybrid and Autotetraploid Breeding Parents. J. Am. Soc. Hortic. Sci..

[73] Mendes B., Mourão Filho F., Farias P., Benedito V. (2001). Citrus Somatic Hybridization with Potential for Improved Blight and CTV Resistance. Vitr. Cell. Dev. Biol.-Plant.

[74] Skoog F., Miller C.O. (1957). Chemical Regulation of Growth and Organ Formation in Plant Tissues Cultured in Vitro. Symp. Soc. Exp. Biol..

[75] Ikeuchi M., Ogawa Y., Iwase A., Sugimoto K. (2016). Plant Regeneration: Cellular Origins and Molecular Mechanisms. Development.

[76] Murashige T. (1974). Plant Propagation Through Tissue Cultures. Annu. Rev. Plant Biol..

[77] Pulianmackal A.J., Kareem A.V.K., Durgaprasad K., Trivedi Z.B., Prasad K. (2014). Competence and Regulatory Interactions during Regeneration in Plants. Front. Plant Sci..

[78] Russell J.A., Ahuja M.R. (1993). Advances in the Protoplast Culture of Woody Plants. Micropropagation of Woody Plants.

[79] Ahuja M.R. (1984). Protoplast Research in Woody Plants. Silvae Genet..

[80] Russell J.A., McCown B.H. (1986). Culture and Regeneration of *Populus* Leaf Protoplasts Isolated from Non-Seedling Tissue. Plant Sci..

[81] Grosser J.W. (1994). Observations and Suggestions for Improving Somatic Hybridization by Plant Protoplast Isolation, Fusion, and Culture. HortScience.

[82] Long Y., Yang Y., Pan G., Shen Y. (2022). New Insights into Tissue Culture Plant-Regeneration Mechanisms. Front. Plant Sci..

[83] Nassour M., Dorion N. (2002). Plant Regeneration from Protoplasts of Micropropagated *Pelargonium* x *Hortorum* ‘Alain’: Effect of Some Environmental and Medium Factors on Protoplast System Efficiency. Plant Sci..

[84] Reed K.M., Bargmann B.O.R. (2021). Protoplast Regeneration and Its Use in New Plant Breeding Technologies. Front. Genome Ed..

[85] Lopez-Arellano M., Dhir S., Albino N., Santiago A., Morris T., Dhir S. (2015). Somatic Embryogenesis and Plantlet Regeneration from Protoplast Culture of *Stevia rebaudiana*. Br. Biotechnol. J..

[86] Tahami S.K., Chamani E. (2018). Efficient Protocol for Protoplast Isolation and Pla Nt Regeneration of *Fritillaria imperialis* L. J. Agric. Sci. Technol..

[87] Prakash M.G., Gurumurthi K. (2010). Effects of Type of Explant and Age, Plant Growth Regulators and Medium Strength on Somatic Embryogenesis and Plant Regeneration in Eucalyptus Camaldulensis. Plant Cell Tissue Organ Cult..

[88] Jie E.-Y., Kim S.-W., Jang H.-R., In D.-S., Liu J.-R. (2011). Myo-Inositol Increases the Plating Efficiency of Protoplast Derived from Cotyledon of Cabbage (Brassica Oleracea Var. Capitata). J. Plant Biotechnol..

[89] Konovalov I.N., Zujkova I.V., Zinov’ev L.S. (1960). Effect of Gibberellic Acid on the Growth and Winterhardiness of Woody Plants. Bot. Zhurnal.

[90] Wang Y., Yao R.L. (2019). Increased Endogenous Indole-3-Acetic Acid:Abscisic Acid Ratio Is a Reliable Marker of *Pinus massoniana* Rejuvenation. Biotech. Histochem..

[91] Saha N., Dutta Gupta S. (2018). Promotion of Shoot Regeneration of *Swertia chirata* by Biosynthesized Silver Nanoparticles and Their Involvement in Ethylene Interceptions and Activation of Antioxidant Activity. Plant Cell Tissue Organ Cult..

[92] Nacheva L.R., Ivanova V. (2017). Silver Nitrate and Chlorhexidine Gluconate—Effective Surface Sterilization Agents in Disinfection Procedures at Initiation of Woody Shoot Tip and Embryo Culture. J. BioSci. Biotechnol..

[93] Pardaz J.E., Ojagh S., Kazemnia H.D. (2015). Effect of Polyvinylpyrrolidone (PVP) on Meristem Establishment and In-Vitro Organogenesis of Iranian Pear (*Pyrus glabra*). Int. J. Agric. Biosci..

[94] Bilska K., Wojciechowska N., Alipour S., Kalemba E.M. (2019). Ascorbic Acid—The Little-Known Antioxidant in Woody Plants. Antioxidants.

[95] Davey M.R., Anthony P., Power J.B., Lowe K.C. (2005). Plant Protoplasts: Status and Biotechnological Perspectives. Biotechnol. Adv..

[96] Deryckere D., Eeckhaut T., Van Huylenbroeck J., Van Bockstaele E. (2012). Low Melting Point Agarose Beads as a Standard Method for Plantlet Regeneration from Protoplasts within the *Cichorium* genus. Plant Cell Rep..

[97] Vardi A., Spiegel-Roy P., Galun E. (1982). Plant Regeneration from Citrus Protoplasts: Variability in Methodological Requirements among Cultivars and Species. Theor. Appl. Genet..

[98] Tomar U.K., Dantu P.K. (2010). Protoplast Culture and Somatic Hybridization. Cellular and Biochemical Science.

[99] Grosser J.W., Calovic M., Louzada E.S. (2010). Protoplast Fusion Technology—Somatic Hybridization and Cybridization. Plant Cell Culture.

[100] Kumari N., Gupta A., Pandey B.C., Kushwaha R., Yaseen M., Mishra M.K., Kumari N. (2023). In Vitro Cultures: Challenges and Limitations. Plants for Immunity and Conservation Strategies.

[101] Lin Y.-C., Li W., Chen H., Li Q., Sun Y.-H., Shi R., Lin C.-Y., Wang J.P., Chen H.-C., Chuang L. (2014). A Simple Improved-Throughput Xylem Protoplast System for Studying Wood Formation. Nat. Protoc..

[102] Ollitrault P., Guo W.W., Grosser J.W. (2007). Somatic Hybridization. Citrus Genetics, Breeding and Biotechnology.

[103] Kuzminsky E., Meschini R., Terzoli S., Pavani L., Silvestri C., Choury Z., Scarascia-Mugnozza G. (2016). Isolation of Mesophyll Protoplasts from Mediterranean Woody Plants for the Study of DNA Integrity under Abiotic Stress. Front. Plant Sci..

[104] Schum A., Hofmann K., Ghalib N., Tawfik A. (2001). Factors Affecting Protoplast Isolation and Plant Regeneration in *Rosa* spp. Gartenbauwissenschaft.

[105] González-Arnao M.T., Dolce N., González-Benito M.E., Castillo Martínez C.R., Cruz-Cruz C.A., Ahuja M.R., Jain S.M. (2017). Approaches for In Vitro Conservation of Woody Plants Germplasm. Biodiversity and Conservation of Woody Plants.

[106] Guan Y., Li S.-G., Fan X.-F., Su Z.-H. (2016). Application of Somatic Embryogenesis in Woody Plants. Front. Plant Sci..

[107] Cui H., Yu Z., Deng J., Gao X., Sun Y., Xia G. (2009). Introgression of Bread Wheat Chromatin into Tall Wheatgrass via Somatic Hybridization. Planta.

[108] Song Y., Luo W., Wu Y., Li X., Albert N.W., Zhang Y., Chen X., Lin-Wang K., Deng C.H., Hu Z. (2023). A Callus-Derived Regeneration and Agrobacterium-Mediated Gene Transformation Developed for Bilberry, Vaccinium Myrtillus. Plant Cell Tissue Organ Cult..

[109] Hu Q., Andersen S., Dixelius C., Hansen L. (2002). Production of Fertile Intergeneric Somatic Hybrids between Brassica Napus and Sinapis Arvensis for the Enrichment of the Rapeseed Gene Pool. Plant Cell Rep..

[110] Harms C.T. (1983). Somatic Incompatibility in the Development of Higher Plant Somatic Hybrids. Q. Rev. Biol..

[111] Feng Q., Xiao L., He Y., Liu M., Wang J., Tian S., Zhang X., Yuan L. (2021). Highly Efficient, Genotype-Independent Transformation and Gene Editing in Watermelon (*Citrullus lanatus*) Using a Chimeric ClGRF4-GIF1 Gene. J. Integr. Plant Biol..

[112] Altpeter F., Springer N.M., Bartley L.E., Blechl A.E., Brutnell T.P., Citovsky V., Conrad L.J., Gelvin S.B., Jackson D.P., Kausch A.P. (2016). Advancing Crop Transformation in the Era of Genome Editing. Plant Cell.

[113] Chen Z., Debernardi J.M., Dubcovsky J., Gallavotti A. (2022). Recent Advances in Crop Transformation Technologies. Nat. Plants.

[114] Maher M.F., Nasti R.A., Vollbrecht M., Starker C.G., Clark M.D., Voytas D.F. (2020). Plant Gene Editing through de Novo Induction of Meristems. Nat. Biotechnol..

[115] Martín-Valmaseda M., Devin S.R., Ortuño-Hernández G., Pérez-Caselles C., Mahdavi S.M.E., Bujdoso G., Salazar J.A., Martínez-Gómez P., Alburquerque N. (2023). CRISPR/Cas as a Genome-Editing Technique in Fruit Tree Breeding. Int. J. Mol. Sci..

[116] Liu L., Qu J., Wang C., Liu M., Zhang C., Zhang X., Guo C., Wu C., Yang G., Huang J. (2024). An Efficient Genetic Transformation System Mediated by Rhizobium Rhizogenes in Fruit Trees Based on the Transgenic Hairy Root to Shoot Conversion. Plant Biotechnol. J..

[117] Lee S.-J., Lee B.H., Jung J.-H., Park S.K., Song J.T., Kim J.H. (2018). GROWTH-REGULATING FACTOR and GRF-INTERACTING FACTOR Specify Meristematic Cells of Gynoecia and Anthers. Plant Physiol..

[118] Wang K.E., Shi L., Liang X., Zhao P., Wang W., Liu J., Chang Y., Hiei Y., Yanagihara C., Du L. (2022). The Gene TaWOX5 Overcomes Genotype Dependency in Wheat Genetic Transformation. Nat. Plants.

[119] Feng M.-Q., Jiang N., Wang P.-B., Liu Y., Xia Q.-M., Jia H.-H., Shi Q.-F., Long J.-M., Xiao G.-A., Yin Z.-P. (2023). miR171-Targeted SCARECROW-LIKE Genes CsSCL2 and CsSCL3 Regulate Somatic Embryogenesis in Citrus. Plant Physiol..

[120] Kusaba M. (2004). RNA Interference in Crop Plants. Curr. Opin. Biotechnol..

[121] Jinek M., Chylinski K., Fonfara I., Hauer M., Doudna J.A., Charpentier E. (2012). A Programmable Dual-RNA–Guided DNA Endonuclease in Adaptive Bacterial Immunity. Science.

[122] Wang P., Li Z., Li H., Zhang D., Wang W., Xu X., Xie Q., Duan Z., Xia X., Guo G. (2024). SMART CROPs. New Crops.

[123] Bai M., Hu X., Lin W., Peng C., Kuang H., Zhong X., Li Y., Chen B., Wang J., Li H. (2024). Development of PmCDA1-Based High-Efficiency Cytidine Base Editors (ChyCBEs) Incorporating a GmRad51 DNA-Binding Domain in Soybean. New Crops.

[124] Gilbert L.A., Horlbeck M.A., Adamson B., Villalta J.E., Chen Y., Whitehead E.H., Guimaraes C., Panning B., Ploegh H.L., Bassik M.C. (2014). Genome-Scale CRISPR-Mediated Control of Gene Repression and Activation. Cell.

[125] Pan C., Li G., Malzahn A.A., Cheng Y., Leyson B., Sretenovic S., Gurel F., Coleman G.D., Qi Y. (2022). Boosting Plant Genome Editing with a Versatile CRISPR-Combo System. Nat. Plants.

[126] Anders C., Hoengenaert L., Boerjan W. (2023). Accelerating Wood Domestication in Forest Trees through Genome Editing: Advances and Prospects. Curr. Opin. Plant Biol..

[127] Lee K., Wang K. (2023). Strategies for Genotype-Flexible Plant Transformation. Curr. Opin. Biotechnol..

[128] Zhang C., Tang Y., Tang S., Chen L., Li T., Yuan H., Xu Y., Zhou Y., Zhang S., Wang J. (2024). An Inducible CRISPR Activation Tool for Accelerating Plant Regeneration. Plant Comm..

[129] Zhang F. (2019). Development of CRISPR-Cas Systems for Genome Editing and Beyond. Q. Rev. Biophys..

[130] Mironova V., Xu J. (2019). A Single-Cell View of Tissue Regeneration in Plants. Curr. Opin. Plant Biol..

[131] Xu M., Du Q., Tian C., Wang Y., Jiao Y. (2021). Stochastic Gene Expression Drives Mesophyll Protoplast Regeneration. Sci. Adv..

[132] Sui J., Tian H., Ding Z., Kong X. (2024). Crop Designs: The Ideal Root Architecture for Future Crop Breeding. New Crops.

[133] Feng W., Gao P., Wang X. (2024). AI Breeder: Genomic Predictions for Crop Breeding. New Crops.

[134] Debernardi J.M., Tricoli D.M., Ercoli M.F., Hayta S., Ronald P., Palatnik J.F., Dubcovsky J. (2020). A GRF–GIF Chimeric Protein Improves the Regeneration Efficiency of Transgenic Plants. Nat. Biotechnol..

[135] Mukami A., Juma B.S., Mweu C., Ngugi M., Oduor R., Mbinda W.M. (2022). Plant Regeneration from Leaf Mesophyll Derived Protoplasts of Cassava (Manihot Esculenta Crantz). PLoS ONE.

[136] Mehrotra S., Goyal V. (2013). Evaluation of Designer Crops for Biosafety—A Scientist’s Perspective. Gene.

[137] Ahmad A., Jamil A., Munawar N. (2023). GMOs or Non-GMOs? The CRISPR Conundrum. Front. Plant Sci..

[138] Wolt J.D., Wang K., Sashital D., Lawrence-Dill C.J. (2016). Achieving Plant CRISPR Targeting That Limits Off-Target Effects. Plant Genome.

